# Precision lung cancer screening from CT scans using a VGG16-based convolutional neural network

**DOI:** 10.3389/fonc.2024.1424546

**Published:** 2024-08-19

**Authors:** Hua Xu, Yuanyuan Yu, Jie Chang, Xifeng Hu, Zitong Tian, Ouwen Li

**Affiliations:** ^1^ Department of Infection Control, Shandong Provincial Hospital Affiliated to Shandong First Medical University, Shandong, Jinan, China; ^2^ Data Science Institute, Shandong University, Jinan, Shandong, China; ^3^ Institute for Medical Dataology, Cheeloo College of Medicine, Shandong University, Jinan, China; ^4^ International Center, Jinan Foreign Language School, Shandong, Jinan, China

**Keywords:** lung cancer screening, medical image recognition, computed tomography scans, VGG16 architecture, convolutional neural network

## Abstract

**Objective:**

The research aims to develop an advanced and precise lung cancer screening model based on Convolutional Neural Networks (CNN).

**Methods:**

Based on the health medical big data platform of Shandong University, we developed a VGG16-Based CNN lung cancer screening model. This model was trained using the Computed Tomography scans data of patients from Pingyi Traditional Chinese Medicine Hospital in Shandong Province, from January to February 2023. Data augmentation techniques, including random resizing, cropping, horizontal flipping, color jitter, random rotation and normalization, were applied to improve model generalization. We used five-fold cross-validation to robustly assess performance. The model was fine-tuned with an SGD optimizer (learning rate 0.001, momentum 0.9, and L2 regularization) and a learning rate scheduler. Dropout layers were added to prevent the model from relying too heavily on specific neurons, enhancing its ability to generalize. Early stopping was implemented when validation loss did not decrease over 10 epochs. In addition, we evaluated the model’s performance with Area Under the Curve (AUC), Classification accuracy, Positive Predictive Value (PPV), and Negative Predictive Value (NPV), Sensitivity, Specificity and F1 score. External validation used an independent dataset from the same hospital, covering January to February 2022.

**Results:**

The training and validation loss and accuracy over iterations show that both accuracy metrics peak at over 0.9 by iteration 15, prompting early stopping to prevent overfitting. Based on five-fold cross-validation, the ROC curves for the VGG16-Based CNN model, demonstrate an AUC of 0.963 ± 0.004, highlighting its excellent diagnostic capability. Confusion matrices provide average metrics with a classification accuracy of 0.917 ± 0.004, PPV of 0.868 ± 0.015, NPV of 0.931 ± 0.003, Sensitivity of 0.776 ± 0.01, Specificity of 0.962 ± 0.005 and F1 score of 0.819 ± 0.008, respectively. External validation confirmed the model’s robustness across different patient populations and imaging conditions.

**Conclusion:**

The VGG16-Based CNN lung screening model constructed in this study can effectively identify lung tumors, demonstrating reliability and effectiveness in real-world medical settings, and providing strong theoretical and empirical support for its use in lung cancer screening.

## 1 Background

Lung cancer is one of the leading causes of cancer-related deaths worldwide, with an estimated 1.8 million deaths, accounting for 18.7% of the total (1–5). Moreover, both the incidence and mortality rates of lung cancer are on the rise, particularly in developing and middle-income countries (6, 7). Given the vast population of China, this implies a large number of new cases and deaths annually, placing immense strain on the healthcare system. In Taiwan, a study assessed low-dose lung computed tomography (CT) screening criteria among Asian ethnic groups, finding that risk-based strategies more effectively identify high-risk non-smokers, emphasizing the need to optimize screening criteria for better early detection (8). Currently, lung CT scans have become a primary tool in modern medicine for early screening of lung diseases, especially lung cancer (9–12). Henschke et al. demonstrated that CT screening can significantly improve the detection rate of early-stage lung cancer compared to traditional screening methods, which is crucial for patient survival rates (13). This further proves the significance of CT screening in the early detection and treatment of lung cancer. However, these place rigorous demands on a physician’s interpretive abilities. To effectively interpret these high-resolution images, physicians must possess extensive clinical medical knowledge and expertise in radiology. Subjectivity and variability in interpretation can lead to inconsistent diagnoses and even misdiagnosis. Therefore, there is a pressing need to seek more advanced methods to assist physicians in achieving more accurate and efficient CT screening interpretations, ultimately enhancing diagnostic outcomes and patients’ medical experiences.

In recent years, deep learning, especially Convolutional Neural Networks (CNN), has been proven to be an incredibly effective tool for automating and optimizing the analysis process of medical imaging. It has also been widely applied in various medical imaging systems such as Magnetic Resonance Imaging (MRI), CT, and X-rays. Litjens et al. conducted in-depth research on how CNN can improve the accuracy of image classification, segmentation, and disease detection (14). Rajpurkar et al. applied a deep learning model called CheXNeXt to chest X-ray images, demonstrating that CheXNeXt’s performance in automatic diagnosis of various chest diseases is comparable to that of practicing radiologists, and in some cases, it even surpasses the performance of human experts (15). These studies provide substantial evidence for the application of deep learning techniques in clinical practice, but still face a series of challenges in areas such as data insufficiency, model overfitting, and interpretability of diagnostic results.

Motivated by the urgent need to improve early lung cancer detection and diagnosis, this study developed a lung cancer screening model based on the CNN architecture. To enhance generalization and reduce overfitting, we employed several strategies. Data augmentation techniques, including random resizing, cropping, horizontal flipping, color jitter, and random rotation, were applied to increase the diversity of the training data. We used five-fold cross-validation to robustly assess performance by ensuring each fold maintained the same ratio of malignant to non-malignant images. The model was fine-tuned with an SGD optimizer (learning rate 0.001, momentum 0.9, and L2 regularization) and a learning rate scheduler. Dropout layers were added to prevent the model from relying too heavily on specific neurons, thus enhancing its ability to generalize. Early stopping was implemented to halt training when validation loss did not decrease over 10 consecutive epochs. In addition, we used a comprehensive set of evaluation metrics, including Area Under the Curve (AUC), Classification accuracy, Positive Predictive Value (PPV), and Negative Predictive Value (NPV), Sensitivity, Specificity and F1 score to ensure the model’s exceptional performance across diverse criteria. Our aim is to leverage the powerful capabilities of CNNs to provide a cutting-edge, accurate, and effective lung cancer screening method, integrating it into daily medical workflows to improve screening accuracy.

## 2 Methods

**Figure 1:**
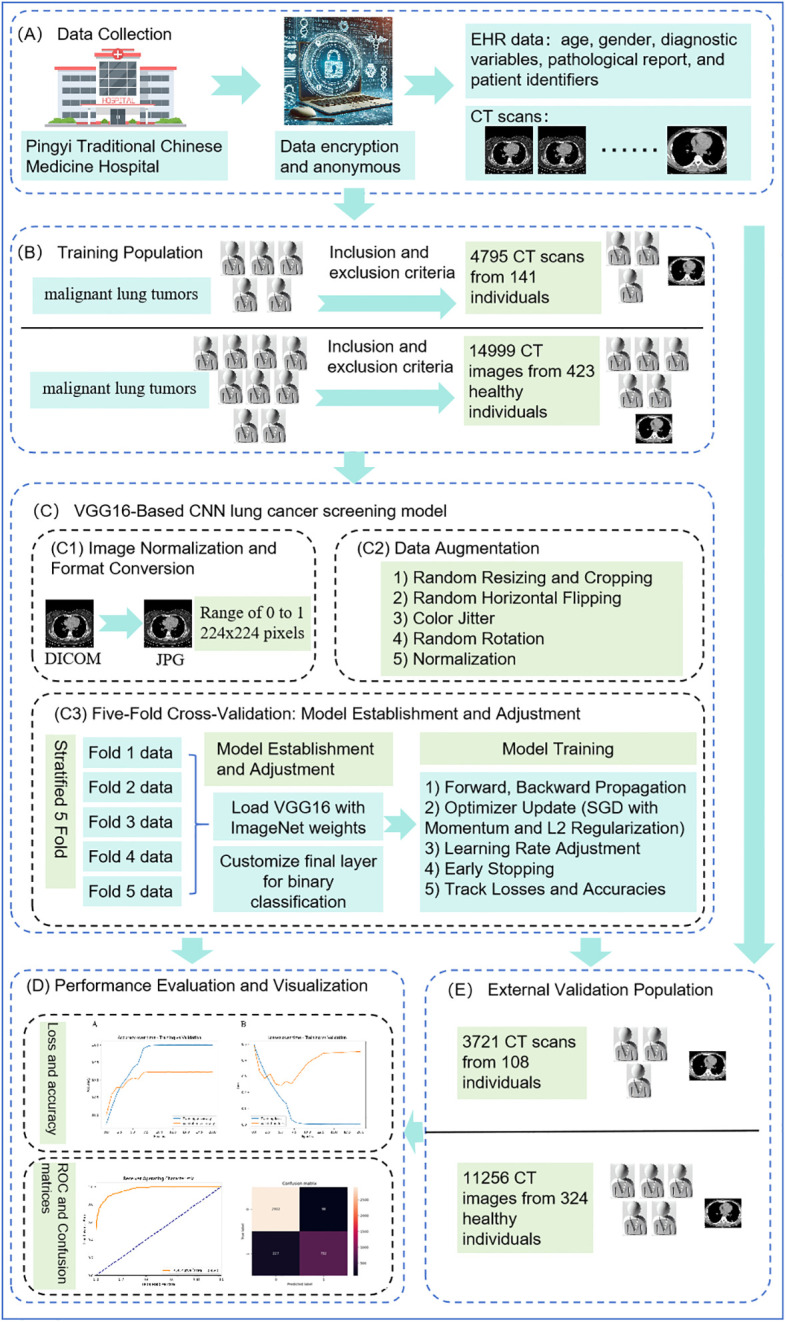
Comprehensive workflow of VGG16-based convolutional neural network. **(A)** shows data collection, **(B)** indicates the process of identifying the training population, **(C)** illustrates the construction of the VGG16-Based CNN lung cancer screening model, **(D)** depicts model evaluation and visualization, and **(E)** represents external validation.

**Figure 1** illustrates the comprehensive workflow of our lung cancer screening model using the VGG16 architecture.

### 2.1 Study population

#### 2.1.1 Training population

##### 2.1.1.1 Construction of the imaging dataset for patients with malignant lung tumors

Based on the health medical big data platform of Shandong University, we gathered a comprehensive dataset from Pingyi Traditional Chinese Medicine Hospital in Shandong Province, including lung CT scans and EHR data such as age, gender, diagnostic variables, pathological reports, and patient identifiers, collected over January and February 2023 (as shown in **Figure 1A**). The pathological report clearly indicated whether the patient had a malignant lung tumor. All patient data were anonymized and collected using encrypted identification numbers to protect privacy and comply with ethical standards. As shown in **Figure 1B**, the inclusion criteria for this study were: 1) Patients with malignant lung tumors who had undergone procedures such as puncture, bronchoscopy, or surgery to confirm the definitive pathological diagnosis. 2) Patients who must have undergone CT scans prior to percutaneous puncture, bronchoscopy biopsy or surgery. 3) Patients who had lung nodules discovered during the CT scan examination. The exclusion criteria were: 1) Patients with concurrent other lung lesions. 2) Patients with a history of lung diseases. 3) Patients with metastatic tumors. Ultimately, a total of 141 patients with malignant lung tumors were included in this study.

##### 2.1.1.2 Construction of the dataset for non-malignant lung tumor patients

For the dataset of non-malignant lung tumor patients, we included same lung CT images and detailed metadata from patients at Pingyi Traditional Chinese Medicine Hospital, collected between January and February 2023. The inclusion criteria required patients to have undergone CT scans. Exclusion criteria (**Figure 1B**), consistent with those for the malignant lung tumor datasets, included patients with concurrent lung lesions, a history of lung diseases, or metastatic tumors. Using a random match method at a ratio of 1:3, a total of 423 non-malignant lung tumor patients were included.

The final dataset was composed of 4795 CT images from 141 individuals with malignant lung tumors and 14999 CT images from 423 individuals with non-malignant lung tumor.

#### 2.1.2 External validation population

An external validation dataset was obtained from Pingyi Traditional Chinese Medicine Hospital in Shandong Province, comprising imaging data collected between January and February 2022 (**Figure 1E**). The inclusion and exclusion criteria were consistent with those used for the primary dataset. This external dataset included 108 patients with malignant lung tumors and 324 patients with non-malignant lung tumors, providing a robust basis for validating the performance of the developed model.

In clinical research, especially with smaller sample sizes, sparse data bias can significantly affect the reliability of statistical estimates. By using a 1:3 random matching design, we increased the sample size of the malignant group, thus reducing the impact of sparse data bias and ensuring more stable and reliable estimates of effect sizes and other statistical parameters (16). In addition, random matching helps to minimize the potential biases that can arise from non-random selection processes. By randomly selecting non-malignant cases at a 1:3 ratio, we aimed to ensure a representative sample of the broader population, thus enhancing the generalizability of our results (17).

Our study utilized both plain and contrast-enhanced CT scans. The choice between these two techniques was based on specific clinical indications and the patient’s condition at the time of imaging, ensuring that the most appropriate diagnostic approach was utilized for each case. The imaging was performed using three different models of CT scanners, including a Philips 256-slice CT, a 64-slice CT, and a 16-slice CT. This diverse array of scanning equipment enabled a comprehensive assessment of the conditions under study, ensuring a robust analysis through varied imaging capabilities.

This study was approved by the Institutional Review Board of Pingyi Traditional Chinese Medicine Hospital in Shandong Province, China. The ethical approval number for our study is PYX2YYYLLWYh2023030602. It is important to note that no informed consent was required from the participants for this study, as only anonymized lung CT images and detailed metadata were used. The anonymization process was rigorously conducted before the data was accessed for research purposes, ensuring that all personal identifiers were removed to protect patient confidentiality.

### 2.2 VGG16-based CNN lung cancer screening model

The Convolutional Neural Network (CNN) is a cornerstone algorithm in deep learning, especially adept at image processing tasks. Since the advent of LeNet in 1998, CNN has become mainstream in computer vision tasks (18). This study attempts to use the VGG16 model to construct a lung cancer screening model. The choice of the VGG16 architecture for lung cancer screening is driven by its distinguished capabilities in handling complex image data and its historical success in diverse image recognition tasks (18). VGG16’s architectural depth and uniformity are ideal for medical imaging, where precision and reliability are paramount. The architecture features 13 convolutional layers that are exceptionally effective at extracting multi-scale features, a fundamental requirement for identifying subtle and critical anomalies in medical images (19, 20). Moreover, VGG16’s robustness and adaptability in processing new and varying datasets make it an exemplary choice for the dynamic requirements of medical diagnostics, as demonstrated by its proven efficacy in diagnosing conditions such as pneumonia from chest X-rays, papillary thyroid carcinomas from cytological images, and brain tumors from MRI scans (15, 21, 22). This combination of deep learning efficiency and versatility underscores why VGG16 is uniquely suited for developing a lung cancer screening model (**Figure 1C**).

#### 2.2.1 Data preparation

##### 2.2.1.1 Image normalization and format conversion

We use *pydicom* to extract DICOM images and normalize their intensity values to a range of 0 to 1, which is essential for consistent CNN performance. Subsequently, we utilize the Figure module from *matplotlib* to display and manipulate image plots, aiding in converting these images to JPG format (23, 24).

#### 2.2.2 Image pre-processing

##### 2.2.2.1 Resizing and normalization

All images were resized to 224x224 pixels to meet the input requirements of the VGG16 model and were normalized to a range of 0 to 1 to standardize the input (19).

##### 2.2.2.2 Data augmentation

To enhance the model’s generalization capabilities and prevent overfitting, data augmentation techniques were applied using the *torchvision.transforms* library. The techniques used included 1) Random Resizing and Cropping: Images were randomly resized and cropped to provide a variety of image sizes and perspectives to the model. 2) Random Horizontal Flipping: Images were randomly flipped horizontally to make the model invariant to left-right orientation. 3) Color Jitter: Random adjustments to brightness, contrast, saturation, and hue to introduce variability. 4) Random Rotation: Images were randomly rotated to make the model invariant to orientation. 5) Normalization: Pixel values were normalized to ensure consistent intensity values across all images (19). By applying these augmentations, we increased the diversity of our training data, prevented overfitting, and ensured that the model could generalize well to unseen data.

#### 2.2.3 Five-fold cross-validation

We implemented the five-fold cross-validation method to reduce the risk of overfitting to a particular subset of the data. This method involved splitting the dataset into five parts, ensuring each fold maintained the same ratio of malignant to non-malignant images. Each fold served as a validation set once, while the remaining four folds constituted the training set (25).

#### 2.2.4 Model establishment and adjustment

We used the VGG16 architecture, which is a well-known deep CNN model pre-trained on the ImageNet dataset. VGG16 consists of 16 layers, including 13 convolutional layers and 3 fully connected layers. Given our task of binary classification (malignant lung tumors vs. non-malignant lung tumors), we modified the final fully connected layer of the VGG16 model to output two classes (19). Specific layers of the pre-trained model were unfrozen to allow fine-tuning during training (26).

#### 2.2.5 Model training

During each iteration, the model underwent forward and backward propagation on the training data. The SGD optimizer, with a learning rate of 0.001 and momentum of 0.9, and weight decay of 0.0005 (L2 regularization), was used to update the model weights. The binary cross-entropy loss function was employed to calculate the loss and both training and validation losses and accuracies were recorded for performance evaluation (27).

#### 2.2.6 Preventing overfitting

Several strategies were employed to prevent overfitting, including: 1) Dropout layers were added to the model to randomly drop neurons during training, which helps in preventing overfitting. 2) A learning rate scheduler was used to dynamically adjust the learning rate during training, helping to fine-tune the model and avoid overfitting. 3) Early stopping was implemented, terminating training if there was no significant decrease in validation loss over 10 consecutive epochs (28).

#### 2.2.7 Performance evaluation and results visualization

Training and validation loss and accuracy were recorded at the end of each epoch. These metrics were plotted to visualize the learning progress and identify potential overfitting or underfitting issues (27). As shown in **Figure 1D**, the ROC curve was plotted to evaluate the trade-off between sensitivity and specificity at various threshold settings (29). The area under the ROC curve (AUC) was calculated to quantify the overall ability of the model to discriminate between positive and negative cases (29). A confusion matrix was generated to provide a detailed breakdown of the model’s predictions and their alignment with the actual outcomes. This matrix helped in understanding the distribution of true positives, true negatives, false positives, and false negatives, which is crucial for evaluating the performance of the classification model (29, 30). At the end of Area Under the Curve (AUC), classification accuracy, Positive Predictive Value (PPV), and Negative Predictive Value (NPV), Sensitivity, Specificity and F1 score were calculated as evaluation metrics (30).

#### 2.2.8 External validation

To ensure the model’s generalizability and robustness, the trained model was further evaluated on the external validation dataset obtained from Pingyi Traditional Chinese Medicine Hospital in Shandong Province (**Figure 1E**). The AUC was computed, and results were visualized using ROC curves to provide a comprehensive evaluation of the model’s performance on an independent dataset.

### 2.3 Software details

The code was written in Python, and executed on the Jupyter server at the Health and Medical Big Data Research Institute of Shandong University.

## 3 Result

### 3.1 General characteristics of patients with malignant lung tumors

A total of 141 patients with malignant lung tumors were included, consisting of 97 males and 44 females. Their ages ranged from 44 years old to 89 years, with a median age of 69 years and an interquartile range (IQR) of 63 to 74 years. Additionally, 423 individuals with no-lung tumors were included, comprising 225 males and 198 females. The age of those without lung tumors ranged from 20 to 95 years, with a median age of 67 years and an IQR of 54.5 to 76.6 years. The frequency distribution of ages for both groups is detailed in **Supplementary Material Tables 1 and 2**.

### 3.2 Results for pulmonary imaging based on CNN

**Figure 2:**
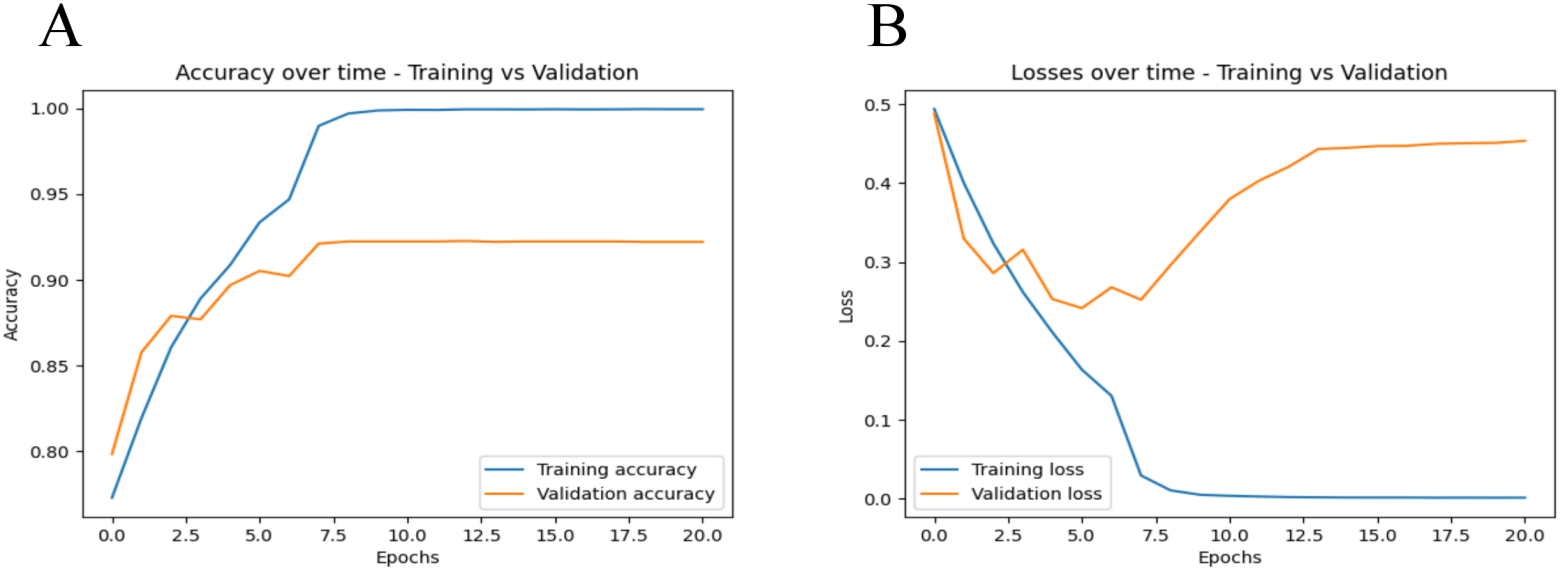
Training and validation metrics over iterations. **(A)** displays the accuracy metrics for both training and validation sets across epochs. The blue line represents the training accuracy, while the orange line represents the validation accuracy. **(B)** shows the loss values for both the training and validation datasets over each training epoch. The blue line represents the training loss, while the orange line represents the validation loss.

**Figure 2** shows the loss and accuracy of the training and validation sets during each iteration. As can be seen from **Figure 2A**, the accuracy of both the training and validation sets generally increases with the number of iterations. By the time the number of iterations reaches 10, the accuracy of both the training and validation sets is at its highest, with both achieving an accuracy of over 90%. As shown in **Figure 2B**, when the number of iterations reaches 15, the program determines that the validation error has not improved for 10 consecutive epochs. Therefore, we chose an iteration count of 15 for early stopping. This early stopping strategy helps prevent overfitting and ensures the model’s robustness by terminating training when no significant improvement in validation loss is observed over 10 consecutive epochs.

**Figure 3:**
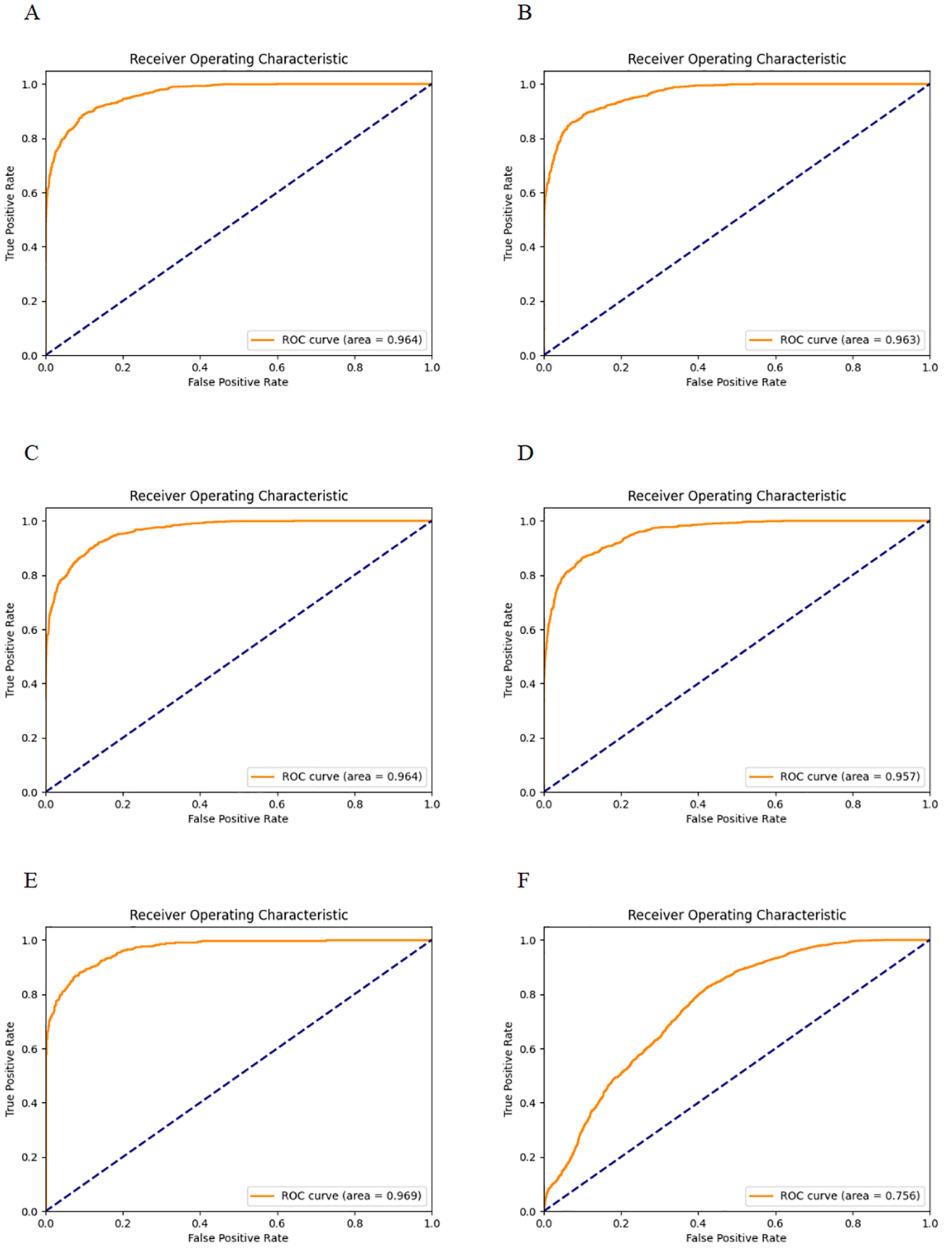
ROC curves for five-fold cross-validation and external validation. **(A–E)** display the ROC curves for each fold of the five-fold cross-validation. **(F)** shows the ROC curve for the external validation dataset.

**Figure 3**- displays the ROC curves for the five-fold cross-validation and the external dataset, while **Table 1** lists the evaluation indices along with their 95% Confidence Intervals (CIs) for five-fold cross-validation. The CNN-based pulmonary imaging diagnostic model consistently demonstrated high diagnostic accuracy, evidenced by an average AUC of 0.963 ± 0.004 across five-fold cross-validation, as depicted in **Figures 3A–E**. This robust performance is presented in the first row of **Table 1**, where each fold’s AUC score approximates 0.96, with narrow 95% confidence intervals. Such consistently high AUC values across multiple validation folds attest to the model’s reliable capability to differentiate between malignant and non-malignant cases.

**Table 1:** The evaluation index and it’s 95% confidence interval for five-fold cross-validation.

Evaluation index[95%CI]	Fold 1	Fold 2	Fold 3	Fold 4	Fold 5
AUC	0.9643 [0.9642, 0.9643]	0.9628[0.9628, 0.9629]	0.9640[0.9639, 0.9640]	0.9566[0.9565, 0.9567]	0.9690[0.9690, 0.9691]
Classification accuracy	0.9179 [0.9089, 0.9261]	0.9179[0.9089, 0.9261]	0.9154[0.9063, 0.9237]	0.9113[0.9021, 0.9198]	0.9222[0.9134, 0.9301]
PPV	0.8819[0.8582, 0.9021]	0.8747[0.8507,0.8953]	0.8537[0.8289,0.8755]	0.8486[0.8233,0.8709]	0.8798[0.8563,0.8999]
NPV	0.9275[0.9178,0.936]	0.9296[0.9201,0.9381]	0.9331[0.9237,0.9414]	0.9291[0.9195,0.9376]	0.9339[0.9246,0.9421]
Sensitivity	0.7633[0.7354, 0.7891]	0.7716[0.7440, 0.7971]	0.7852[0.7581, 0.8100]	0.7716 [0.7440, 0.7971]	0.7862[0.7592, 0.8110]
Specificity	0.9673[0.9604, 0.9731]	0.9647[0.9574, 0.9707]	0.9570[0.9491, 0.9637]	0.9560[0.9481, 0.9628]	0.9657 [0.9585, 0.9716]
F1 score	0.8183[0.8058, 0.8298]	0.8199[0.8076, 0.8316]	0.8180 [0.8056, 0.8296]	0.8083[0.7957, 0.8202]	0.8304[0.8182, 0.8416]

Additionally, the confusion matrices for all 5 runs are provided in the **Supplementary Material Figures 1-5**. Based on the confusion matrix, the classification accuracy, PPV and NPV were 0.917 ± 0.004, 0.868 ± 0.015, and 0.931 ± 0.003, respectively, with each fold’s accuracy, PPV, and NPV along with their 95% CIs, detailed in lines two to four of **Table 1**. Classification accuracy measures the overall effectiveness of the model in correctly identifying both positive and negative cases, with a reported accuracy of approximately 91.1%. PPV specifically assesses the accuracy of the model’s positive predictions, indicating that approximately 86.6% of the lung cancer diagnoses made by the model are accurate. An NPV of 93.1% ensures a high probability that negative diagnoses are correct, minimizing the risk of missed diagnoses. Together, high PPV and NPV highlight the model’s reliability, making it suitable for clinical lung cancer screening. According to the confusion matrix of the five-fold cross-validation, the Sensitivity, Specificity and F1 score were 0.776 ± 0.01, 0.962 ± 0.005 and 0.819 ± 0.008, respectively, with these results for each fold shown in lines five to seven of **Table 1**. The sensitivity of 77.6% indicates a robust capability to detect true cases of lung cancer, effectively minimizing the risk of missing diagnoses (false negatives). The specificity of 96.2% demonstrates the model’s precision in identifying individuals who do not have lung cancer, significantly reducing the occurrence of false positives. Additionally, an F1 score of 81.9% reflects a well-balanced trade-off between precision and sensitivity, ensuring the model’s overall accuracy and reliability in medical diagnostics. This comprehensive validation across multiple metrics confirms the model’s effectiveness in lung cancer detection.

The external validation showed that our model maintained robust performance (**Figure 3F**), with an AUC and 95%CI of 0.7564 [0.7563, 0.7564]. The result confirms the model’s ability to generalize well across different patient populations and imaging conditions.

## 4 Discussion

Lung cancer has become a significant public health problem in China, with rising incidence and mortality rates as highlighted in the “Cancer incidence and mortality in China, 2016” report and recent data from the American Cancer Society (ACS) (31, 32). With the advancement of technology and medicine, radiological lung screening has become a pivotal means for early detection and evaluation of pulmonary diseases, especially lung cancer. Despite extensive research and practice, existing methods still have limitations. The survival rate for patients with advanced lung cancer remains low (33, 34), and early diagnosis and treatment are becoming increasingly important (35–37). Aberle et al. have pointed out that compared to traditional chest X-rays, lung cancer screenings using low-dose CT can significantly reduce lung cancer mortality rates (38). This pivotal discovery laid a solid scientific foundation for the promotion and application of low-dose CT screening (39–41). However, traditional imaging analysis methods have certain limitations, such as high missed diagnosis rate, misdiagnosis rate, and limited ability to interpret intricate images (42, 43). Therefore, with the increasing complexity and resolution of medical imaging, there’s a growing reliance on advanced computer-aided diagnostic systems to assist in understanding and interpreting these images (44–46). Deep learning techniques, especially CNNs, have revolutionized medical imaging analysis (47).

Our study constructed a precise lung cancer screening model based on CNN. Initial preprocessing and data augmentation ensured the quality and consistency of lung cancer imaging data. Overfitting was mitigated through multiple strategies including data augmentation techniques such as random resizing and cropping, random horizontal flipping, color jitter, and random rotation. L2 regularization was applied via weight decay in the optimizer, and a learning rate scheduler dynamically adjusted the learning rate during training. Dropout layers were added to the model to prevent reliance on specific neurons, and early stopping was implemented to halt training if validation loss did not decrease over 10 consecutive epochs. Five-fold cross-validation demonstrated the model’s robustness. Our study achieved an AUC of 0.963 ± 0.004 on the validation set, indicative of excellent diagnostic capability. This high AUC value demonstrated the model’s robust capacity to distinguish accurately between lung cancer and those without, minimizing both false positives and false negatives. These performances are consistent with a classification accuracy of 0.917 ± 0.004. Additionally, the PPV, NPV, Sensitivity, Specificity, and F1 score were 0.868 ± 0.015, 0.931 ± 0.003, 0.776 ± 0.01, 0.962 ± 0.005, and 0.819 ± 0.008, respectively. These performance metrics collectively demonstrate the model’s high degree of diagnostic accuracy and reliability in identifying lung cancer. The classification accuracy of 91.7% indicates a strong overall ability to correctly classify cases as either having lung cancer or not, supported by a high PPV (86.8%) and NPV (93.1%), which ensure that the positive and negative diagnoses made by the model are likely correct. The sensitivity of 77.6% shows that the model is capable of identifying a substantial majority of true positive cases, critical for early and accurate disease detection. Furthermore, the specificity of 96.2% underscores the model’s effectiveness in correctly ruling out disease in healthy individuals, reducing the likelihood of unnecessary treatments. Finally, an F1 score of 81.9% reflects a balanced trade-off between precision and recall, validating the model’s utility in clinical settings where both detecting cases and avoiding false alarms are equally important. Together, these metrics not only underline the model’s capability but also highlight its potential to significantly enhance patient management and treatment outcomes in clinical practice.

Lu et al. created a CNN model for predicting the long-term incidence of lung cancer, achieving an AUC of 74.9% (48). Ardila et al. reached an AUC of 94.4% using a 3D CNN model for lung nodule detection (49). Cellina et al. reviewed numerous studies on AI applications in lung cancer imaging and diagnosis, reporting AUC values ranging from 87% to 95% (50, 51). This is slightly lower than the AUC of 96.3% achieved by our model, indicating superior performance in distinguishing between lung cancer and non-cancer cases. Additionally, different models reported classification accuracies typically around 90% to 97%, with sensitivity and specificity values ranging from 75% to 95%. Our model’s classification accuracy of 91.7% and sensitivity of 77.6% fall within this range. However, our model’s sensitivity is slightly lower, suggesting a need for improvement in detecting true positive cases. In contrast, our model’s specificity of 96.2% is significantly higher than other studies, indicating its superior ability to correctly identify individuals without lung cancer, minimizing false positives. Comparing positive predictive value (PPV), Hsu et al. achieved 15.0%, whereas our study reported a much higher PPV of 86.8%, reflecting greater accuracy in positive predictions and ensuring fewer false positives. Both studies demonstrated high negative predictive values (NPV), with Hsu et al. at 99.0% and our study at 93.1%, highlighting effectiveness in correctly predicting negative cases (51). Overall, our model’s robust performance metrics indicate its potential to significantly enhance lung cancer screening and diagnostic accuracy in clinical settings.

The data collection period from January to February 2023 at Pingyi Traditional Chinese Medicine Hospital was relatively short, which may limit the diversity of our dataset and affect the generalizability of our model. To address this issue, we employed extensive data augmentation techniques, such as random resizing, cropping, horizontal flipping, color jitter, random rotation, and normalization. Despite these efforts, it remains essential to increase the sample size to enhance the model’s generalizability. We have planned additional data collection efforts and propose implementing a continuous learning framework to periodically retrain the model with updated data from our hospital and datasets from various regions and medical institutions, ranging from community clinics to large tertiary hospitals. This approach aims to enhance the model’s adaptability to variations in clinical practice and its generalizability.

In addition, using data from the same institution collected at different times for external validation may not be ideal. However, existing literature supports the effectiveness of temporal external validation (52, 53), which led us to choose this approach for our study. In addition, the external validation confirmed an AUC of 0.7564 across various patient populations and imaging conditions, which is significantly lower than the training model’s AUC of 0.963. The two cohorts, although originating from the same institution, represent different time periods. This temporal difference could potentially affect the model’s performance, influenced by changes in patient demographics, CT scanning protocols, or other clinical practices over time. Despite implementing several strategies during model training, such as dropout and L2 regularization, the generalizability of our model across different geographical regions and medical institutions remains a significant challenge. To address this, we outline planned future studies intended to apply our model to datasets collected from different regions, including both urban and rural settings, and from various types of medical institutions ranging from community clinics to large tertiary hospitals. This will allow us to assess its performance and adaptability in diverse healthcare environments.

We observed an increase in validation loss after the 7th epoch, as shown in **Figure 2**. To enhance the model’s generalization and prevent overfitting, we have implemented various measures, including data augmentation, dynamic adjustment of learning rates, integration of dropout layers and L2 regularization and so on. However, the behavior of machine learning models often remains influenced by inherent characteristics of the training data, such as latent noise and complex nonlinear relationships. These factors may cause the model to quickly adapt to these characteristics in the early stages of training, which could pose challenges as training progresses. Despite extensive efforts to prevent overfitting, the model may still exhibit heightened sensitivity to certain specific features of the training data, especially after prolonged training periods. Looking forward, we plan to further enhance our model’s performance by expanding our dataset and exploring the use of updated and more sophisticated model architectures.

In practical clinical applications, deep learning models, including ours, have shown remarkable potential for improving the accuracy and speed of medical diagnoses, as supported by research from Erickson et al. and others (54). Our CNN-based model demonstrates high accuracy in the early detection and diagnosis of lung cancer, offering the potential for continuous improvement through updates and retraining with new data. In addition, challenges such as data privacy, model interpretability, and acceptance within the medical community remain. Therefore, therefore, future work will also need to focus on strengthening data privacy measures and enhancing the interpretability of the model. Addressing these challenges comprehensively will be key to fully integrating advanced diagnostic tools like ours into clinical practice, ultimately improving early lung cancer diagnosis and patient outcomes.

## Data Availability

The datasets presented in this article are not readily available because the data is individual-level data restricted for sharing due to ethical requirements. Requests to access the datasets should be directed to apocalypse1523@163.com.
