# Supplementary material for: Precision lung cancer screening from CT scans using a VGG16-based convolutional neural network
Source: Front Oncol. 2024 Aug 19;14:1424546. doi: 10.3389/fonc.2024.1424546 (PMC11369893; doi:10.3389/fonc.2024.1424546)
Supplement: Supplementary file 1 [file Datasheet1.docx]

**1 Supplemental Material Table 1.** **Age Group of Lung Cancer**

Supplemental Material Table 1 Age group of lung cancer

| Age group | Frequency | Percentage |
| --- | --- | --- |
| 40~50 | 6 | 4.255% |
| 50~60 | 21 | 14.894% |
| 60~70 | 45 | 31.915% |
| 70~80 | 56 | 39.716% |
| >80 | 13 | 9.220% |
| Sum | 141 | 100% |

**2 Supplemental Material Table 2. Age group of Non-****Lung Cancer**

Supplemental Material Table 1 Age group of lung cancer

| Age group | Frequency | Percentage |
| --- | --- | --- |
| <40 | 26 | 6.1465% |
| 40~50 | 43 | 10.165% |
| 50~60 | 75 | 17.731% |
| 60~70 | 99 | 23.404% |
| 70~80 | 107 | 25.296% |
| >80 | 73 | 17.258% |
| Sum | 423 | 100% |

**3 Supplemental Material Figures 1-5. Confusion matrices for all 5-Fold Cross-Validation**

**
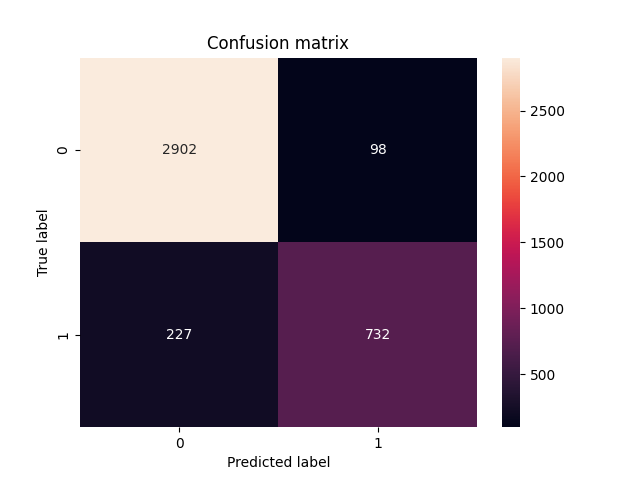
**

**Supplemental Material Figure 1.** Confusion Matrix of the VGG16-CNN Model (First Fold). This matrix displays the classification performance of the VGG16-CNN model during the first fold of a five-fold cross-validation process. The x-axis represents the predicted labels with '0' indicating negative and '1' indicating positive predictions. The y-axis displays the true labels. Each cell shows the count of predictions made by the model: the top left cell (2902) represents true negatives (correctly predicted negative cases), the top right cell (98) represents false positives (negative cases incorrectly predicted as positive), the bottom left cell (227) represents false negatives (positive cases incorrectly predicted as negative), and the bottom right cell (732) represents true positives (correctly predicted positive cases).

**
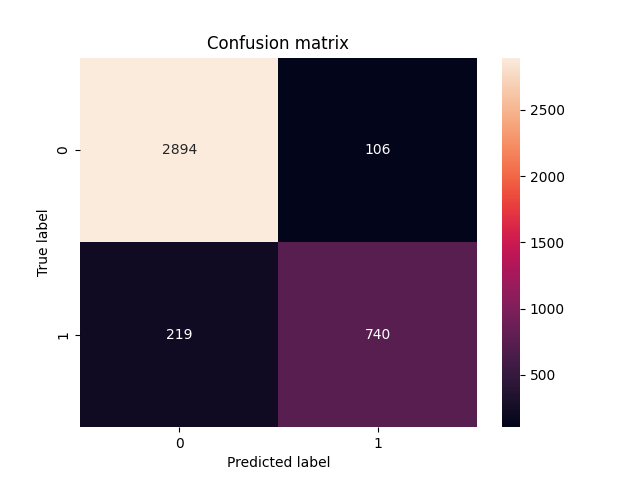
**

**Supplemental Material Figure 2.** Confusion Matrix of the VGG16-CNN Model (Second Fold). This matrix illustrates the classification performance during the second fold of a five-fold cross-validation process. The x-axis represents the predicted labels with '0' for negative and '1' for positive predictions, while the y-axis shows the true labels. The cell values indicate the count of predictions: top left (2894) for true negatives, top right (106) for false positives, bottom left (219) for false negatives, and bottom right (740) for true positives.

**
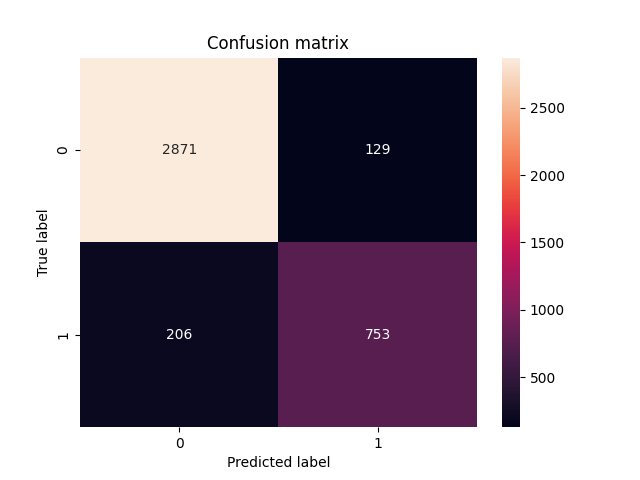
**

**Supplemental Material Figure 3.** Confusion Matrix of the VGG16-CNN Model (Third Fold of Five-Fold Cross-Validation) displays the classification accuracy, with the x-axis indicating predicted labels (0 for negative, 1 for positive) and the y-axis showing true labels. The matrix values are as follows: true negatives (2871), false positives (129), false negatives (206), and true positives (753).

**
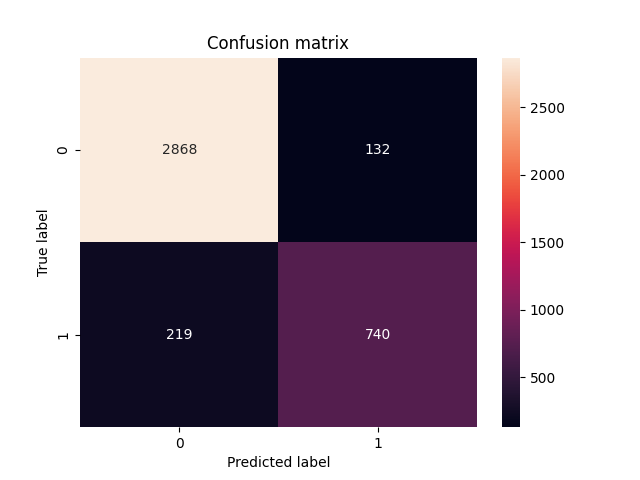
**

**Supplemental Material Figure 4.** Confusion Matrix of the VGG16-CNN Model (Fourth Fold of Five-Fold Cross-Validation) displays the classification accuracy, with the x-axis indicating predicted labels (0 for negative, 1 for positive) and the y-axis showing true labels. The matrix values are as follows: true negatives (2868), false positives (132), false negatives (219), and true positives (740).

**
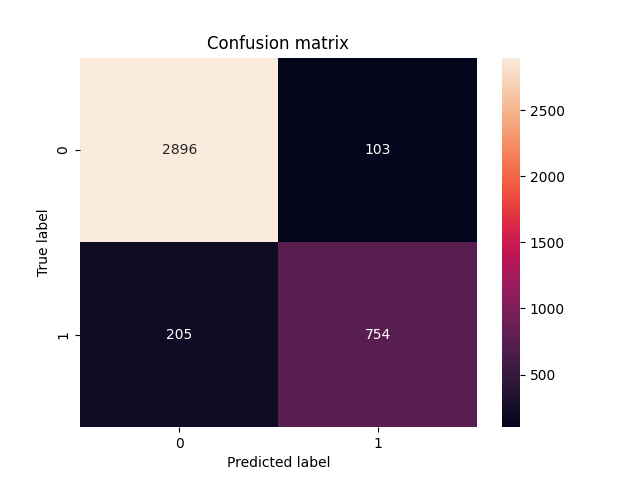
**

**Supplemental Material Figure 5.** Confusion Matrix of the VGG16-CNN Model (Fourth Fold of Five-Fold Cross-Validation) displays the classification accuracy, with the x-axis indicating predicted labels (0 for negative, 1 for positive) and the y-axis showing true labels. The matrix values are as follows: true negatives (2896), false positives (103), false negatives (205), and true positives (754).
